# Grassland Resistance and Resilience after Drought Depends on Management Intensity and Species Richness

**DOI:** 10.1371/journal.pone.0036992

**Published:** 2012-05-16

**Authors:** Anja Vogel, Michael Scherer-Lorenzen, Alexandra Weigelt

**Affiliations:** 1 Institute of Ecology, Friedrich Schiller University Jena, Jena, Germany; 2 Michael Scherer-Lorenzen, Faculty of Biology – Geobotany, University of Freiburg, Freiburg, Germany; 3 Alexandra Weigelt, Institute of Biology, University of Leipzig, Leipzig, Germany; Umea University, Sweden

## Abstract

The degree to which biodiversity may promote the stability of grasslands in the light of climatic variability, such as prolonged summer drought, has attracted considerable interest. Studies so far yielded inconsistent results and in addition, the effect of different grassland management practices on their response to drought remains an open question. We experimentally combined the manipulation of prolonged summer drought (sheltered vs. unsheltered sites), plant species loss (6 levels of 60 down to 1 species) and management intensity (4 levels varying in mowing frequency and amount of fertilizer application). Stability was measured as resistance and resilience of aboveground biomass production in grasslands against decreased summer precipitation, where resistance is the difference between drought treatments directly after drought induction and resilience is the difference between drought treatments in spring of the following year. We hypothesized that (i) management intensification amplifies biomass decrease under drought, (ii) resistance decreases with increasing species richness and with management intensification and (iii) resilience increases with increasing species richness and with management intensification.

We found that resistance and resilience of grasslands to summer drought are highly dependent on management intensity and partly on species richness. Frequent mowing reduced the resistance of grasslands against drought and increasing species richness decreased resistance in one of our two study years. Resilience was positively related to species richness only under the highest management treatment. We conclude that low mowing frequency is more important for high resistance against drought than species richness. Nevertheless, species richness increased aboveground productivity in all management treatments both under drought and ambient conditions and should therefore be maintained under future climates.

## Introduction

There is agreement that the world's ecosystems will likely have to cope with future climatic changes, such as increased mean temperatures, a higher frequency of extreme weather events as well as changes in wind and precipitation patterns [1]. Among the different scenarios are projected decreases in summer precipitation and increases in autumn, winter and spring precipitation in subtropical and temperate regions (see also [2] that shows the same for the region of our study site). Along with the present and ongoing climate change, biodiversity is challenged by land-use changes to meet the growing demand for ecosystem services [3]. The consequences of those changes for ecosystem functioning, ecosystem services and human wellbeing have been the focus of research in the last few decades. It has been found that plant species diversity can have positive effects on multiple ecosystem processes [4] and if many times, places, functions and environmental changes were considered [5]. Aboveground plant biomass production, the most-studied process in biodiversity research, has been consistently found to rise in response to plant diversity in grasslands [6], [7], [8], [9], [10], an important finding for agricultural management. This positive relationship of species richness and productivity even holds under nutrient-rich conditions [11], [12] and perturbations such as intense livestock grazing [13].

Beyond productivity itself, the temporal stability of biomass production has also been found to relate positively with species richness [14], [15], [16], [17], meaning that temporal variability in productivity is lower in species-rich compared to species-poor communities. Besides the consistent results of diversity effects on temporal stability, the relationship between diversity and other aspects of stability, like resistance and resilience after perturbations (such as climatic changes) are mixed. Resistance, the degree of change after perturbations [18], is usually calculated as the difference of some performance measure between perturbed and unperturbed conditions and reflects the extent to which the mean of an ecosystem property changes after a single perturbation event. Resilience (in the sense of engineering resilience [19]), the time after perturbation until pre-perturbation levels are regained [18], is usually expressed as the rate of return of a variable at a given time after perturbation [20], [21], [22]. Both, resistance and resilience after perturbations were expected to increase with species richness [14]. In experimental studies it has been found to be true for resistance of biomass production against parasitism [23] and herbivore attack [24], but also neutral relationships between herbivore resistance and species richness have been reported [13], [25]. The experimental results on resistance of grassland biomass production against drought show mostly negative or neutral relationships with species richness [21], [22], [26], depending on the level of productivity. It has been documented that more productive grasslands have a lower ability to withstand perturbation (lower resistance) than less productive communities, indicated by the higher loss of absolute aboveground biomass after a drought perturbation [22]. Thus, depending on the slope of the biodiversity-productivity relationship under unperturbed conditions, resistance either decreases with increasing species richness (negative slope) [21], [22], increases with species richness (positive slope, which was only documented for a grassland experiment, where species richness was not independently manipulated) [26] or does not change (no significant relationship), when species richness has no effect on productivity like in the studies by Kahmen *et al.* [27] and Wang *et al.* [28]. It can thus be hypothesized that high species richness has a positive effect on the productivity of ecosystems but also results in a lower ability to resist future climatic changes. Besides the important ecosystem property to resist perturbations, a fast return to preperturbation levels (high resilience) would help to maintain ecosystem functioning. In very early studies it had been suggested that resistance and resilience are inversely correlated [29]. MacGillivray *et al.* [30] supported this prediction by measuring drought resistance and resilience in semi-natural grassland communities. It is also consistent with the work by van Ruijven *et al.* [22] who reported about experimental grasslands with decreasing resistance with species richness and increasing resilience. Although they confirmed that resistance rather depends on initial productivity of grasslands than on species richness *per se* (proportional resistance did not decrease with species richness), they found species richness to be a stronger predictor for recovery than productivity (recovery and proportional recovery increased with species richness).

Despite these findings, it remains unclear whether the idea that species richness promotes the productivity of grasslands at the consequence of lower resistance but higher resilience against perturbation, is applicable to a broad range of grasslands. For example, Gilgen and Buchmann [31] found that drought effects on biomass production vary between different grassland types along an altitudinal gradient. However, the explanatory factors of that study, namely environmental conditions and management strategies, were not independent and their individual effects could thus not be separated. In managed grasslands, management intensity has profound consequences on species richness and productivity. Fertilized grasslands, for example, have low species richness but a high productivity due to the high resource availability. Because of a low resistance with high productivity, one would expect that fertilization decreases the resistance of grasslands to perturbation. Indeed, Grime *et al.* [32] suggested that fertile grasslands would be less resistant compared to extensively managed grasslands because they contain more species with a high relative growth rate, which are highly susceptible to drought stress. However, higher growth rates should lead to faster regrowth after perturbation and we would expect a high resilience in fertilized grasslands.

**Figure 1:**
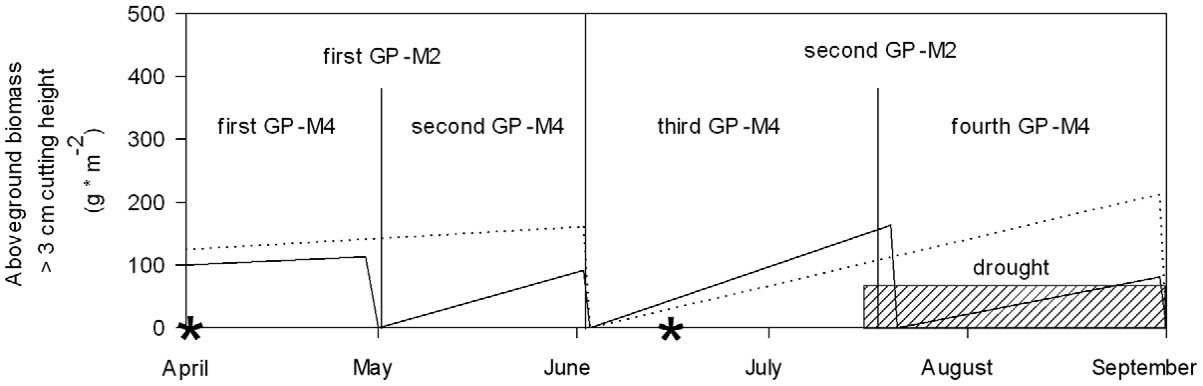
Time course of the growing season, including management and drought interventions of our study system. Stars indicate fertilization dates, vertical lines show mowing dates and dashed area represents our drought period. Frequently mown grassland types (M4, solid line) had four cuts per year and therefore four growth periods (first to fourth GP) previous to every cut. Normal mown grassland types (M2, dotted line) had two cuts per year and therefore two growth periods. Aboveground biomass is plotted as mean standing biomass above cutting height for each mowing grassland type.

We experimentally manipulated plant species richness, management intensity (amount of fertilizer and mowing frequencies, Figure 1) and prolonged summer drought separately to investigate the effects of biodiversity on resistance and resilience against drought perturbation in grasslands differing in their management intensity. We hypothesize that (i) biomass decreases under drought perturbation, especially under high management intensity due to the higher productivity under fertilization, (ii) resistance decreases with increasing species richness and with management intensification but proportional resistance (measure of resistance that is corrected for initial biomass productivity) does not change with species richness and (iii) resilience as well as proportional resilience increases with increasing species richness as well as with management intensity.

## Results

**Figure 2:**
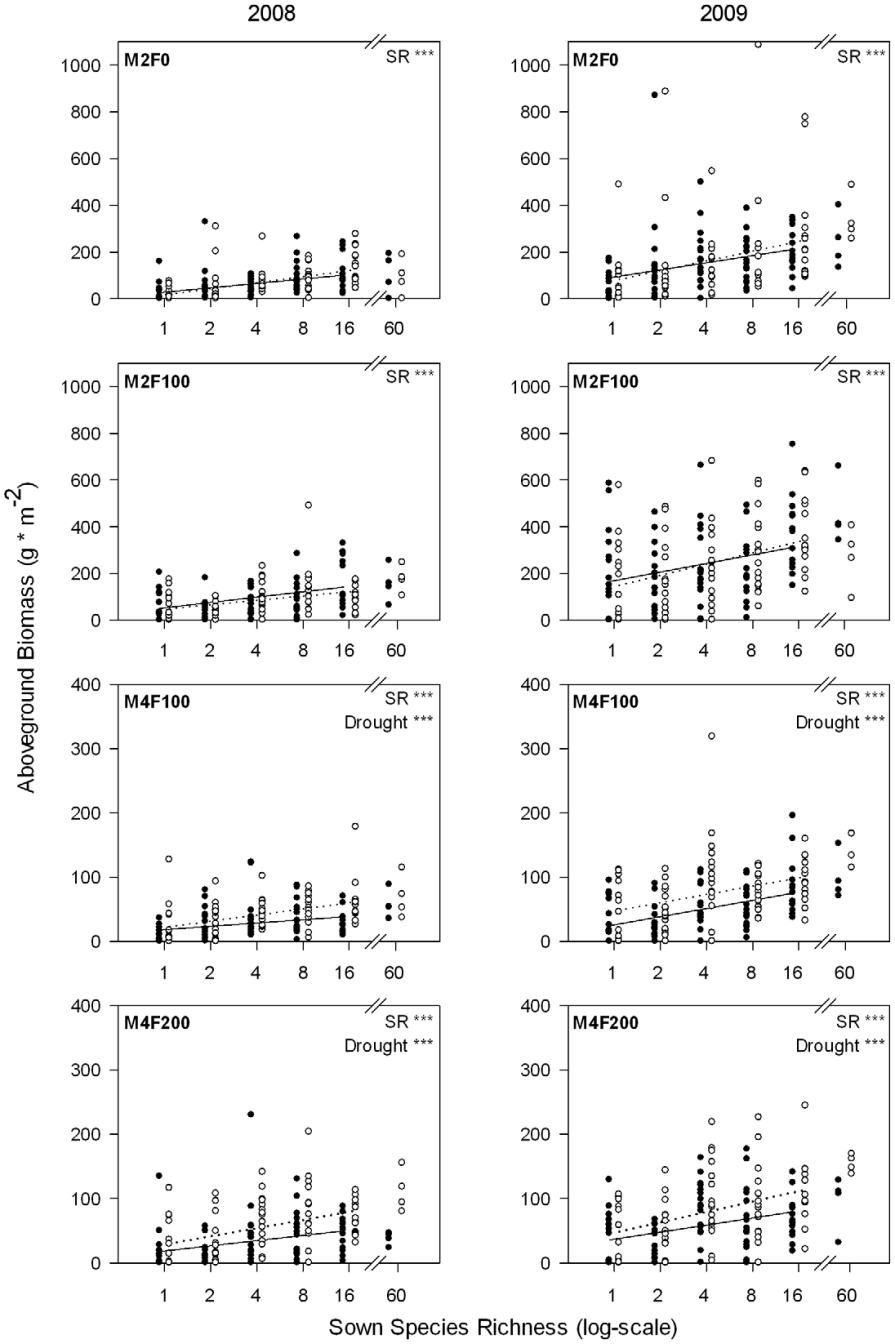
Aboveground biomass across sown species richness gradient for each management treatment. Aboveground biomass at the end of the induced drought period in August 2008 (left column) and 2009 (right column) measured regrowth since the last cut (M2-types: June; M4-types: July). The ambient treatment is given in open circles (dotted regression line) and drought treatment in closed circles (solid regression line). Significant effects obtained from mixed models for every single management treatment per year: SR = effect of sown species richness (linear), drought = difference in drought and ambient treatments, * = p<0.05, ** = p<0.01, *** = p<0.001.

**Figure 3:**
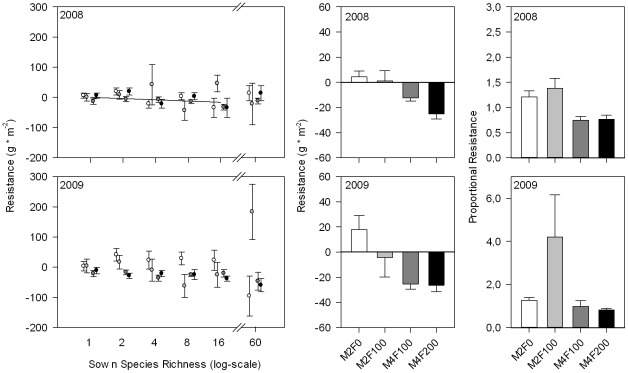
Resistance of biomass production for 2008 and 2009. Resistance was calculated as the difference of drought and corresponding ambient treatments at the end of the drought period in August and was plotted against species richness (left) and management treatments (middle). Regression lines are given for significant effects of species richness. Proportional resistance (ratio of drought to ambient treatment) was plotted against management treatment (right). Management treatments are shown in white (M2F0), gray (M2F100), dark gray (M4F100) and black (M4F200).

**Figure 4:**
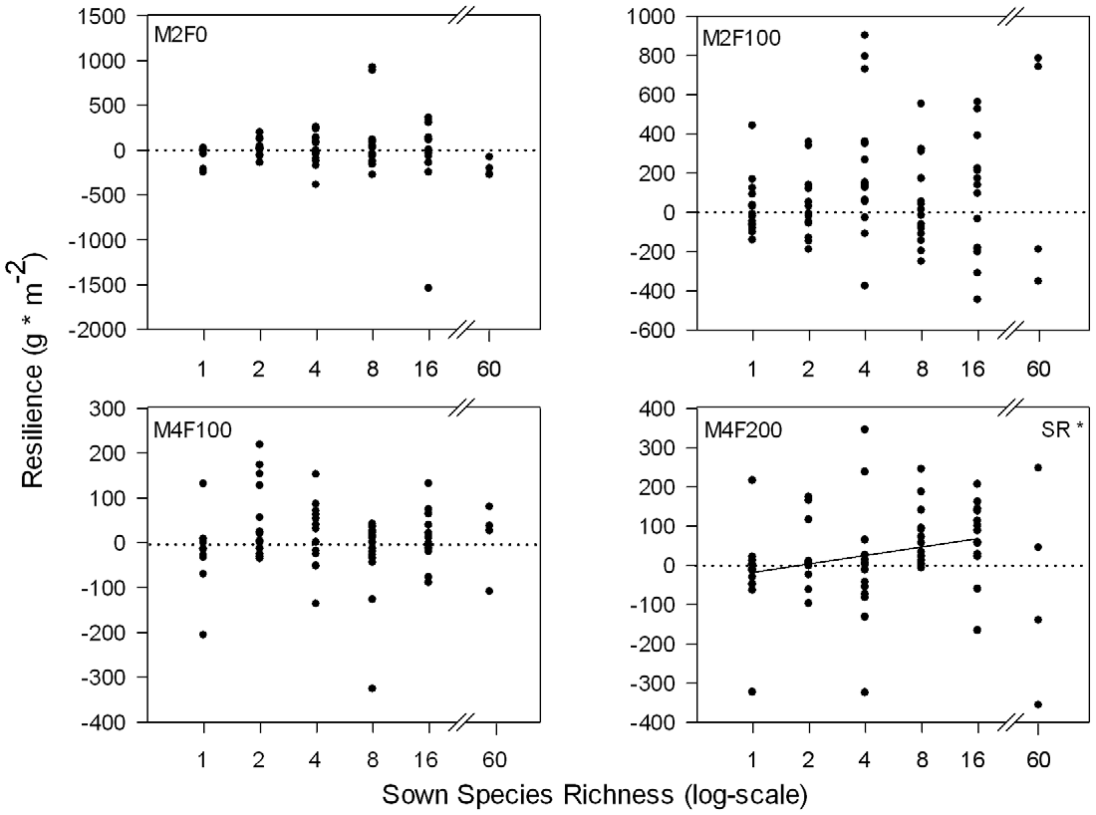
Resilience of biomass over sown species richness for each management treatment. Resilience of biomass was calculated as the difference of drought and corresponding ambient treatments for the first harvest in spring 2009 (M2-types: June, M4-types: April). Regression lines are given for significant effects obtained from linear models for every single management treatment per year: SR = effect of sown species richness (linear), drought = difference in drought and ambient treatments, * = p<0.05, ** = p<0.01, *** = p<0.001.

The harvest at the end of the induced drought period showed significant increases in aboveground biomass with sown species richness in both years (Table 1, Figure 2). This was true for all management and drought treatments. Aboveground biomass was significantly lower under drought only in the frequently mown grasslands (M4F100, M4F200, Figure 2), i.e., the drought response of grasslands was affected by management intensity (Table 1). Resistance slightly decreased with increasing species richness in 2008 (Table 2, Figure 3 left) and with increasing mowing frequency in both years (Table 2, Figure 3). Proportional resistance was not affected by species richness but still decreased with increasing mowing frequency in 2008 (Table 2, Figure 3). We found no effect of the interaction of species diversity and management treatment (mowing frequency or amount of fertilizer) on resistance or proportional resistance. Resilience was positively related to species richness only in the most intensively managed grassland (significant interaction of SR and F in Table 3, Figure 4). Proportional resilience was not affected by species richness (Table 3).

**Table 1:** Summary of mixed effects models for aboveground biomass in August 2008 and 2009 to test for effects of diversity (numbers of initially sown species and functional group richness), management and drought treatments.

	Biomass August 2008					Biomass August 2009			
	df	AIC	L ratio	*p*		AIC	L ratio	*p*	
Nullmodel	11	1882.024				2058.232			
Block	14	1885.305	2.719	0.4370		2061.191	3.041	0.3854	
Species Richness (log-scale) = SR	15	1843.338	43.967	<0.0001	***	2026.597	36.595	<0.0001	***
Number of Functional Groups = FG	16	1845.331	0.007	0.9336		2026.316	2.280	0.1310	
Management	19	1815.300	36.031	<0.0001	***	1941.106	91.210	<0.0001	***
Drought	20	1789.755	27.545	<0.0001	***	1927.831	15.275	0.0001	***
Management×Drought	23	1786.773	8.982	0.0295	*	1930.126	3.705	0.2951	
Management×SR	26	1789.410	3.363	0.3390		1935.555	0.572	0.9029	
Management×FG	29	1793.380	2.030	0.5662		1937.716	3.838	0.2795	
Drought×SR	30	1793.947	1.433	0.2313		1939.645	0.072	0.7890	
Drought×FG	31	1794.484	1.463	0.2264		1941.613	0.032	0.8579	
SR×FG	32	1795.201	1.283	0.2574		1942.251	1.362	0.2431	

Models were fitted by stepwise inclusion of variables and likelihood ratio tests (L ratio) were applied to assess statistical significance of variables (p-values). Significance is given with * = *p*<0.05. ** = *p*<0.01. *** = *p*<0.001; df = degrees of freedom.

**Table 2:** Summary of mixed effects models for resistance and proportional resistance of aboveground biomass after drought in August 2008 and 2009 to test for effects of diversity (numbers of initially sown species and functional group richness), management (separated into mowing and fertilizer amounts) and drought treatments.

		Resistance 2008				Resistance 2009					proportional Resistance 2008				proportional Resistance 2009		
	df	AIC	L ratio	*p*		AIC	L ratio	*p*		df	AIC	L ratio	*p*		AIC	L ratio	*p*
Nullmodel	3	1026.667				1158.463				6	914.183				1025.004		
Block	6	1028.312	4.355	0.2256		1157.890	6.573	0.0868		9	917.457	2.726	0.4358		1028.257	2.746	0.4324
Species richness (log-scale) = SR	7	1025.371	4.941	0.0262	*	1157.731	2.598	0.1417		10	918.179	1.278	0.2582		1030.178	0.080	0.7778
Number of functional groups = FG	8	1025.530	1.840	0.1749		1159.167	0.563	0.4529		11	919.132	1.047	0.3062		1032.016	0.161	0.6880
Mowing = M	9	1007.267	20.263	<0.0001	***	1145.509	15.658	0.0001	***	12	909.735	11.397	0.0007	***	1030.359	3.657	0.0558
M×SR	10	1007.980	1.287	0.2565		1147.369	0.141	0.7077		13	911.734	0.001	0.9757		1032.358	0.001	0.9788
M×FG	11	1008.535	1.445	0.2293		1149.119	0.250	0.6169		14	910.415	3.320	0.0685		1034.324	0.034	0.8534
Fertilizer amount = F	12	1010.209	0.325	0.5685		1150.820	0.298	0.5851		15	911.540	0.874	0.3498		1036.322	0.002	0.9626
F×SR	13	1011.601	0.608	0.4354		1151.880	0.940	0.3322		16	913.419	0.122	0.7272		1038.275	0.047	0.8290
F×FG	14	1010.700	2.901	0.0885		1152.796	1.084	0.2978		17	912.595	2.824	0.0929		1039.751	0.524	0.4689

Models were fitted by stepwise inclusion of variables and likelihood ratio tests (L ratio) were applied to assess statistical significance of variables (p-values). Significance is given with * = *p*<0.05, ** = *p*<0.01, *** = *p*<0.001; df = degrees of freedom.

**Table 3:** Summary of mixed effects models for resilience and proportional resilience of aboveground biomass of the first cut in spring 2009 to test for effects of diversity (numbers of initially sown species and functional group richness), management (separated into mowing and fertilizer amounts) and drought treatments.

		Resilience Spring 2009				proportional Resilience 2009		
	df	AIC	L ratio	*p*		AIC	L ratio	*p*
Nullmodel	6	3898,242				1024,902		
Block	9	3903,177	1,065	0.7855		1026,706	4,196	0.2410
Species richness (log-scale) = SR	10	3903,771	1,406	0.2357		1026,624	2,081	0.1491
Number of functional groups = FG	11	3905,674	0,097	0.7557		1028,398	0,226	0.6345
Mowing = M	12	3905,225	2,449	0.1176		1029,770	0,628	0.4280
M×SR	13	3907,219	0,006	0.9395		1030,613	1,157	0.2821
M×FG	14	3909,208	0,011	0.9161		1032,446	0,167	0.6826
Fertilizer amount = F	15	3907,540	3,668	0.0555		1032,955	1,491	0.2221
F×SR	16	3904,600	4,939	0.0263	*	1033,658	1,297	0.2548
F×FG	17	3905,130	1,470	0.2253		1033,873	1,784	0.1816

Models were fitted by stepwise inclusion of variables and likelihood ratio tests (L ratio) were applied to assess statistical significance of variables (p-values). Significance is given with * = *p*<0.05, ** = *p*<0.01, *** = *p*<0.001; df = degrees of freedom.

## Discussion

In our study we independently manipulated biodiversity loss, management intensity and drought. We were therefore able to distinguish the effects of all single treatments from their interactions on aboveground biomass. We found that the response of experimental grasslands to drought depends on management intensity. Aboveground biomass decreased after induced summer drought only in grasslands with frequent mowing (four times per year), not in grasslands with only two cuts per year. Differences in growth status due to mowing may explain our findings. The low canopy height after mowing generally increases soil surface evaporation through increased wind speed at ground level and low plant cover. Hu et al. [33] reported increased evaporation with decreased canopy density, measured as leaf area index (LAI), especially at LAI values lower than 2 m^2^ * m^−2^. After mowing, LAI in our experiment was close to 0 m^2^ * m^−2^ (data not shown). In addition to reduced precipitation this would mean a further decrease of soil moisture in the frequently mown (M4-) treatment compared to the normal mown (M2-) treatment. Consequently, a decrease of aboveground biomass only occurs if drought hits the communities at an early growth status, when soil is not sufficiently covered by plants. Thus, grasslands with high mowing frequency and hence frequently low LAI have to be considered as more sensitive to drought. In contrast, extensively mown grasslands would only suffer from drought if it occurs in the regrowth phase after mowing. This was not the case in both of the study years for our grasslands mown only twice a year (M2). They were well-advanced in height growth at the beginning of the drought treatment. The same might have been the case in the study of Jentsch et al. [34], where no effect on aboveground productivity could be detected when drought was induced during peak growing season in June [34]. In contrast Pfisterer and Schmid [21] found negative drought effects in their grassland with the same management as in our experiment (two cuts per year and drought induction before late cut). The longer drought period (and therefore shorter time for normal regrowth) compared to our experiment could be one explanation for contrasting results but also site specific (climatic conditions) and treatment differences have to be taken into account. This study site has mean annual precipitation amounts twice as high as in our site (Table 4) and rain shelters were adjusted close to vegetation, thus a stronger heat effect could be assumed.

**Table 4:** Climatic parameters measured on field site during the two study years 2008 and 2009 with reference period 1961–1990 measured by the German Weather Service DWD in Jena, city center.

Month	Air temperature (°C)			Precipitation (mm)			Soil moisture (Vol%)	
	1961–90	2008	2009	1961–90	2008	2009	2008	2009
J	0.40	5.00	−3.09	37.00	24.50	9.00	37.29	22.11
F	1.40	3.76	1.15	34.00	20.40	33.70	37.15	33.31
M	4.80	5.11	5.04	43.00	55.80	42.50	37.97	37.01
A	8.60	7.90	11.58	57.00	91.80	73.70	37.35	31.39
M	13.40	14.05	13.89	62.00	22.00	62.60	25.62	31.05
J	16.70	17.13	15.01	75.00	54.40	52.90	21.74	28.58
J	18.20	18.51	18.34	52.00	40.60	85.10	17.75	31.29
A	17.40	17.90	18.59	63.00	58.60	14.60	16.61	22.34
S	14.20	12.05	14.56	42.00	50.00	53.60	21.71	23.67
O	9.80	9.13	8.42	39.00	55.30	47.30	26.30	28.70
N	5.00	5.60	8.06	41.00	19.90	68.30	28.16	34.00
D	1.70	1.28	0.64	42.00	38.60	80.00	30.59	36.22
Year	9.30	11.75	11.48	587.00	531.90	623.30	28.19	29.97

Values represent monthly means (temperature, soil moisture) or sums (precipitation).

Along with our second hypothesis we wanted to test, whether the observed relationships of resistance and species richness [22] change with management intensity. We only found slightly decreasing resistance with species richness and this effect of species richness did not change with management intensity. Instead we found a strong effect of management intensity itself on resistance because high mowing frequency decreased the resistance of grasslands against drought. High mowing frequency resulted in both lower absolute biomass accumulation (resistance) as well as lower relative biomass accumulation under drought (proportional resistance) compared to normal mown stands. Our results support the idea that species richness affects resistance due to increasing productivity with species number, and not due to number of species *per se* [22], because proportional resistance was not related to species richness. De Boeck *et al.* [35] found that more productive and species-rich communities have a higher evapotranspiration and water use efficiency compared to monocultures. They concluded that decreased aboveground biomass is one potential mechanism for saving water, because it reduces the transpirational surface of the canopy.

After the second drought in the following year, resistance was constant across the plant diversity gradient, meaning that there was no stronger decrease in absolute amounts of aboveground biomass in the species rich compared to the species poor communities due to drought. Different precipitation patterns might explain this year-to-year changes, since rainfall just before the induced drought period was different in both years (Table 4). The main rain events of 2008 occurred in the very wet April, whereas May and June were unusually dry. Soil moisture (volumetric water content measured in 8 cm depth of an unsheltered reference area) decreased to an average of 17.0% in summer. In contrast in 2009, rain events were regularly distributed and average soil moisture was at 25.3% in summer. A higher soil moisture at the beginning of the drought treatment in 2009 may have stimulated a better growth of the plants compared to the year before and might have more rapidly lowered the loss of soil water through evaporation. Species-rich communities are thought to be more water efficient [35], [36], i.e. they produce more biomass per unit of water. Such communities could therefore benefit from higher soil moisture compared to species-poor communities. In consequence, they could be proportionally less affected from drought stress than species poor communities. Whether annual precipitation patterns would explain the different resistance-species richness patterns between years, can only be underpinned by long-term data.

Resilience increased with species richness as previously reported [22] but only under highest management intensity. In contrast, we found that species richness only affected absolute resilience, whereas the proportion of biomass increase in previously dried subplots compared to their ambient conditions (proportional resilience) did not change with species richness. Furthermore, resilience and species richness were only positively related in the very intensively managed grasslands (frequently mown and high fertilized). The positive relationship of resilience and species richness in the M4F200 management treatment was weak but significant and might be strongly due to the positive responses of the 8-species-mixtures. It is known that species richness increases shoot-root-ratios indicating a better resource use with species richness [37]. We can only speculate why this was more effective in the previously dried subplots compared to the corresponding ambient treatment under highest management intensity. It may be explained in part by higher fertilizer amounts affecting belowground processes. It has been reported that drought did not necessarily increase root growth [38], [39], [40]. Together with a decreased aboveground biomass, drought may have decreased shoot-root-ratios especially in more diverse mixtures. The corresponding ambient treatments might have a much higher shoot-root-ratio due to the higher biomass and the well-known fact that fertilization decreases root growth relative to shoot growth. After drought, this lower shoot-root-ratio of the dried subplots may be a prerequisite for better aboveground biomass allocation in comparison to the ambient in the intensively fertilized treatment. Furthermore, the plasticity and intensity of growth responses of roots and shoots under drought is highly species-specific [39], [41], and thus plant species composition is an important determinant of community root and shoot growth [31]. This could explain that especially the 8-species-mixtures had a high resilience, when intensively managed.

Our results indicate that management intensity affects the resistance of grasslands after drought, with growth phenology being the underlying cause: grasslands at the regrowth stage are more sensitive to decreased precipitation and loose more biomass, than grasslands at later stages with a more fully developed canopy. As a consequence, low mowing frequency enhances drought resistance because of a lower probability to face reduced precipitation during the regrowth stage. Nevertheless, species richness and aboveground biomass were positively related even under drought conditions, which shows that biomass yield is higher the more diverse a community is, no matter under which management intensity and climatic conditions the community grew. Thus high plant species diversity should be maintained under future climates.

## Materials and Methods

### Study site and experimental treatments

We used the gradient of plant species richness established in the Jena biodiversity experiment and superimposed a gradient of management intensity and a drought treatment. The field site is located in the floodplain of the river Saale in Jena (Thuringia, Germany, 50°55′N, 11°35′E, 130 m above sea level) with a mean annual air temperature of 9.3°C and precipitation amount of 587 mm measured during 1961–1990. The study site was used as a highly fertilized arable field before [42]. The soils are loamy Eutric Fluvisols. In 2002, 80 grassland plots of different plant species mixtures were established from a pool of 60 mesophilic grassland species from Molinio-Arrhenateretea meadows typical for the regional alluvial plains.

The gradient in plant species richness (1, 2, 4, 8, 16 and 60 species) in the Jena experiment is combined with a gradient in the number of functional groups (1, 2, 3 or 4 functional groups namely grasses, small herbs, tall herbs and legumes) with about four replicates per species richness×functional groups combination. Mixtures were arranged in a randomized block design to account for edaphic variations with increasing distance to the river Saale. Experimental plots were maintained by weeding blockwise in two annual weeding campaigns. For further details see [42].

The gradient in management intensity was established in 2006 with four subplots on every plot of the 80 plant mixtures. Management varied in mowing regime (M2: two cuts, M4: four cuts per year) and the amount of NPK-fertilizer application (F0: no fertilizer; F100: 100.0 kg N ha^−1^ a^−1^, 43.6 kg P ha^−1^ a^−1^, 83.0 kg K ha^−1^ a^−1^; F200: 200 kg N ha^−1^ a^−1^, 87.2 kg P ha^−1^ a^−1^, 166.0 kg K ha^−1^ a^−1^) and was combined as follows: M2F0, M2F100, M4F100, M4F200 [11]. All three fertilizer treatments were arranged randomly on an area of each 1.6 m×4 m within the main plots of 20×20 m, while M2F0 treatment was always located in the central core area of the plots, representing the standard management of the whole field site. Fertilization was done twice a year (31 March and 23 June 2008, 31 March and 16 June 2009) and mowing was done in spring (end of April, only M4-subplots), in early summer (beginning of June, all subplots), end of July (M4-subplots) and in late summer at the beginning of September (all subplots).

Drought was induced in 2008 and 2009 using transparent rain shelters during six weeks in summer previous to the last annual cut (25 July to 2 September 2008 and 16 July to 1 September 2009, Figure 1). Rain shelters were made of LDPE greenhouse film (www.dm-folien.com) in 2008 and of PVC sheets (www.paruschke-kunststoffe.de, product code: PVCSPK7018K10) in 2009 because of its higher durability. Rain shelters were inclined in a height of 1.3 to 1.5 m to enable ventilation and runoff of rain water in one direction 1 m away from our core area. Control subplots remained unsheltered and received ambient precipitation. We established one sheltered (hereafter named “drought” treatment) and one unsheltered subplot (named “ambient”) of 1.6 m×2 m size for each management treatment in each of the 80 plots covering the whole diversity gradient. Measurements of the soil water content revealed a soil moisture decrease of about 17% in the drought treatment compared to the ambient treatment. The open-side construction of the rain shelters could not prevent a temperature increase of about 1.5–2.2°C on soil surface in the drought treatments, but no warming was detected at 20 cm height. The PVC sheets reduced photosynthetically active radiation (PAR) by 28% maximum. In 2009 we established an additional roof control in all plots, e.g. a sheltered subplot where we added collected rain water, to measure the pure roof effect (heat, altered light conditions) on our response variable. We found that the results of the roof control were similar to those of the ambient treatment (data not shown).

Since the Jena Experiment was established, climatic conditions were measured by a weather station directly on field site so that we were able to document weather data during our experimental phase 2008–2009 (Table 4). Rain shelters excluded 59.5 mm precipitation in 2008 (reduction of 40% compared to unroofed subplots during the summer July–September) and 53.7 mm in 2009 (reduction of 35%).

### Data collection

All measurements were restricted to a central area of 1 m×1 m on every subplot to minimize edge effects (precipitation, varying soil nutrients, different height of neighboring vegetation). We clipped aboveground biomass of a 20 cm×50 cm area at 3 cm height above soil surface two days prior to every mowing event of a subplot. Biomass was sorted into sown species, unsown weeds and dead plant material, dried until constant weight (70°C, 48 h) and weighed. Here we present the results of the biomass of sown species.

### Statistical analysis

We calculated resistance and resilience from our biomass data according to van Ruijven and Berendse [22]. In contrast to that study, which compared perturbed and unperturbed plots in two consecutive years, we were able to compare data from each drought treatment with its corresponding ambient treatment at the same time. Resistance of biomass production was calculated as the difference of biomass under perturbed and unperturbed conditions (drought - ambient) at the end of the drought period in August. Proportional resistance calculated as the ratio of drought to ambient treatment biomass was determined to account for productivity effects on resistance. Resilience determines the change in biomass production after perturbation and was calculated as difference of post-drought biomass and the corresponding ambient treatment from the first harvest after drought (M4-subplots: April 2009, M2-subplots: June 2009). Proportional resilience was calculated as the ratio of post-drought biomass and the corresponding ambient treatment from the first harvest after drought. The proportional values indicate, whether the ratio of biomass decrease or increase due to drought change.

We analyzed the data with mixed effects models using the *nlme*-package of R 2.8.1. to account for the nested design of our experiment (drought/ambient nested within management nested within plots of different diversity levels). Because we were not interested in effects of each species combination, we used plots as random factors in the model, as well as management and our drought treatment. We fitted a series of models by stepwise inclusion of fixed effects. First, we included block in our fixed term to account for all edaphic variation in the field and the blockwise management and data sampling. Then we included sown species richness and functional group richness as diversity factors and our experimental treatments (management, drought) and their interaction with diversity treatments stepwise in the fixed term of the models with the maximum likelihood method. We applied likelihood ratio tests for model comparison and estimating the significance of the fixed effects. We are aware that sown diversity and realized diversity can vary between the management treatments, because management intensification is expected to reduce species diversity [43]. We therefore fitted additional models with realized species richness and realized functional group richness instead of sown species and functional group richness. These models presented the same conclusions as with design variables. For better comparison with other experimental results, we present results of sown diversity effects in the paper and realized diversity effects in the (Table S1, S2, S3, Figure S1, S2, S3 and Methods S1 for information on data acquisition of realized species richness). To meet the assumptions of mixed effects models (normally distributed within group errors and random effects), biomass and resistance were log transformed. When data were heteroscedastic (in case of resilience), variance functions were included [44]. The 60 species mixtures merely serve as reference plots and are excluded from analysis as they are not fully compatible with the experimental design of the experiment.

## Supporting Information

Methods S1 Realized species richness was recorded during drought period in August 2008 and 2009 in every subplot. We recorded presence and absence of every single sown species in 10 squares of 1 dm^2^ size along one transect and repeated this three times within our study area of 1 m^2^. The realized species number was the sum of all species that were present in at least one out of the 30 squares.
(DOC)

Figure S1 **Aboveground biomass across realized species richness gradient for each management treatment.** Aboveground biomass at the end of the induced drought period in August 2008 (left) and 2009 (right) for each management treatment since the last cut (M2-types: June; M4-types: July). The ambient treatment is given in open circles (dotted regression line) and drought treatment in closed circles (solid regression line). Significant effects obtained from mixed models for every single management treatment per year: SR = effect of realized species richness (linear), drought = difference in drought and ambient treatments, * = p<0.05, ** = p<0.01, *** = p<0.001.
(TIF)

Figure S2 **Resistance in biomass production over realized species richness.** Resistance was calculated as the difference of drought and corresponding ambient treatments. Realized species richness represents the mean of realized species numbers of drought and ambient treatment. Management treatments are shown in white (M2F0), gray (M2F100), dark gray (M4F100), black (M4F200).
(TIF)

Figure S3 **Resilience of biomass over realized species richness for each management treatment.** Resilience was calculated as the difference of drought and corresponding ambient treatments for the first harvest in spring 2009 (M2-types: June, M4-types: April). Realized species richness represents the mean of realized species numbers of drought and ambient treatment. Regression lines are given for significant effects obtained from linear models for every single management treatment per year: SR = effect of realized species richness (linear), drought = difference in drought and ambient treatments, * = p<0.05, ** = p<0.01, *** = p<0.001.
(TIF)

Table S1 **Summary of mixed effects models for aboveground biomass in August 2008 and 2009 to test for effects of management, drought and diversity (realized numbers of species and functional groups) treatments.**
(DOC)

Table S2 **Summary of mixed effects models for resistance and proportional resistance of aboveground biomass after drought in August 2008 and 2009 to test for effects of diversity (realized numbers of species and functional groups) and management treatments (separated into mowing and fertilizer amounts).**
(DOC)

Table S3 **Summary of mixed effects models for resilience computed as the difference between previously drought and ambient treatment in aboveground biomass as well as for proportional resilience of the first cut in spring 2009 to test for effects of management (separated into mowing and fertilizer amounts) and diversity (realized numbers of species and functional groups) treatments.**
(DOC)
