# Supplementary material for: Grassland Resistance and Resilience after Drought Depends on Management Intensity and Species Richness
Source: PLoS One. 2012 May 16;7(5):e36992. doi: 10.1371/journal.pone.0036992 (PMC3353960; doi:10.1371/journal.pone.0036992)
Supplement: Methods S1 — Realized species richness was recorded during drought period in August 2008 and 2009 in every subplot. We recorded presence and absence of every single sown species in 10 squares of 1 dm2 size along one transect and repeated this three times within our study area of 1 m2. The realized species number was the sum of all species that were present in at least one out of the 30 squares. (DOC) [file pone.0036992.s001.doc]

Methods S1:

Realized species richness was recorded during drought period in August 2008 and 2009 in every subplot. We recorded presence and absence of every single sown species in 10 squares of 1 dm² size along one transect and repeated this three times within our study area of 1 m². The realized species number was the sum of all species that were present in at least one out of the 30 squares.
