# Supplementary figures and images for: Grassland Resistance and Resilience after Drought Depends on Management Intensity and Species Richness
Source: PLoS One. 2012 May 16;7(5):e36992. doi: 10.1371/journal.pone.0036992 (PMC3353960; doi:10.1371/journal.pone.0036992)

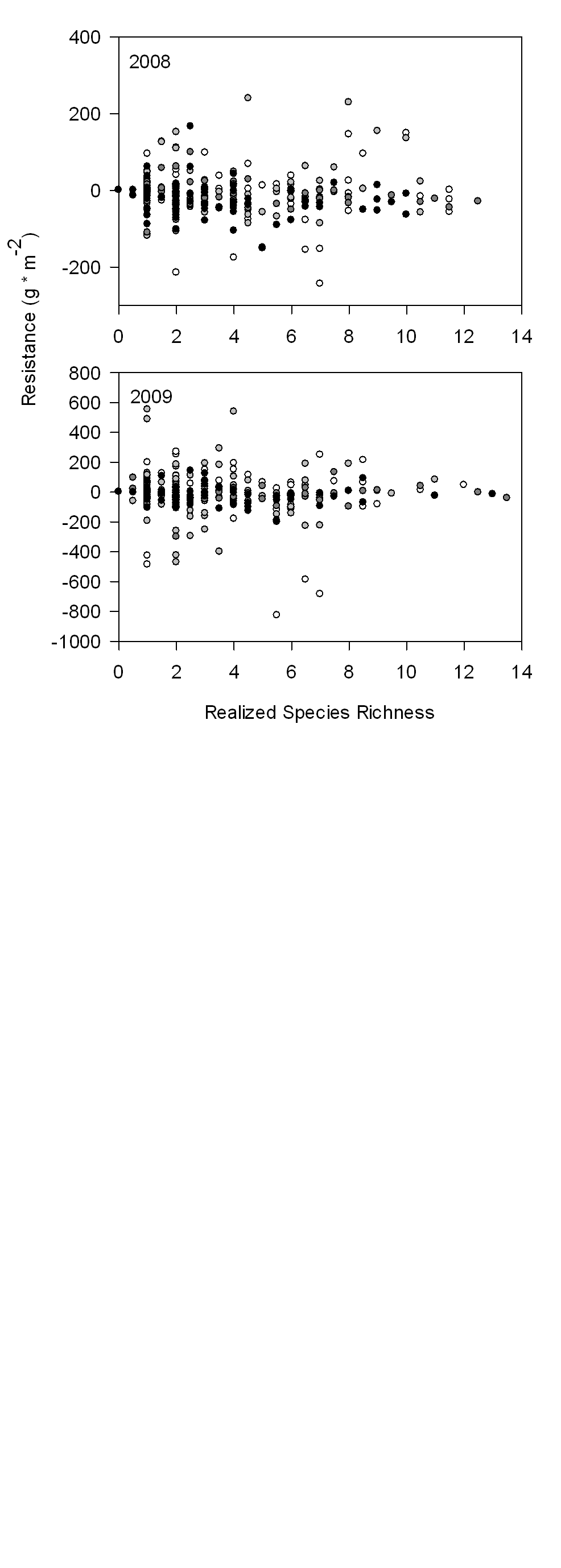

Supplement: Figure S2 — Resistance in biomass production over realized species richness. Resistance was calculated as the difference of drought and corresponding ambient treatments. Realized species richness represents the mean of realized species numbers of drought and ambient treatment. Management treatments are shown in white (M2F0), gray (M2F100), dark gray (M4F100), black (M4F200). (TIF) [file pone.0036992.s003.tif]
