# Supplementary material for: Grassland Resistance and Resilience after Drought Depends on Management Intensity and Species Richness
Source: PLoS One. 2012 May 16;7(5):e36992. doi: 10.1371/journal.pone.0036992 (PMC3353960; doi:10.1371/journal.pone.0036992)
Supplement: Table S1 — Summary of mixed effects models for aboveground biomass in August 2008 and 2009 to test for effects of management, drought and diversity (realized numbers of species and functional groups) treatments. (DOC) [file pone.0036992.s005.doc]

**Table S1:** Summary of mixed effects models for aboveground biomass in August 2008 and 2009 to test for effects of management, drought and diversity (realized numbers of species and functional groups) treatments.

|  | Biomass August 2008 | | | | | Biomass August 2009 | | | |
| --- | --- | --- | --- | --- | --- | --- | --- | --- | --- |
|  | *df* | AIC | L ratio | *p* |  | AIC | L ratio | *p* |  |
| Nullmodel | 11 | 1882.024 |  |  |  | 2058.232 |  |  |  |
| Block | 14 | 1885.305 | 2.719 | 0.4370 |  | 2061.191 | 3.041 | 0.3854 |  |
| Realized Species Richness = RSR | 15 | 1856.362 | 30.942 | <0.0001 | *** | 2036.751 | 26.441 | <0.0001 | *** |
| Realized Number of Functional Groups = RFG | 16 | 1856.860 | 1.503 | 0.2203 |  | 2021.864 | 16.887 | <0.0001 | *** |
| Management | 19 | 1826.150 | 36.710 | <0.0001 | *** | 1933.651 | 94.213 | <0.0001 | *** |
| Drought | 20 | 1804.536 | 23.614 | <0.0001 | *** | 1922.303 | 13.348 | 0.0003 | *** |
| Management x Drought | 23 | 1801.960 | 8.576 | 0.0355 | * | 1925.314 | 2.989 | 0.3934 |  |
| Management x RSR | 26 | 1804.605 | 3.355 | 0.3401 |  | 1929.095 | 2.219 | 0.5281 |  |
| Management x RFG | 29 | 1810.184 | 0.421 | 0.9359 |  | 1931.145 | 3.950 | 0.2669 |  |
| Drought x RSR | 30 | 1812.004 | 0.180 | 0.6715 |  | 1932.531 | 0.614 | 0.4334 |  |
| Drought x RFG | 31 | 1813.718 | 0.285 | 0.5932 |  | 1934.303 | 0.228 | 0.6332 |  |
| RSR x RFG | 32 | 1805.752 | 9.967 | 0.0016 | ** | 1877.262 | 59.041 | <0.0001 | *** |

Models were fitted by stepwise inclusion of variables and likelihood ratio tests (L ratio) were applied to assess statistical significance of variables (p-values). Significance is given with * = *p*<0.05, **= *p*<0.01, *** = *p*<0.001; df = degrees of freedom.
