# Supplementary material for: Grassland Resistance and Resilience after Drought Depends on Management Intensity and Species Richness
Source: PLoS One. 2012 May 16;7(5):e36992. doi: 10.1371/journal.pone.0036992 (PMC3353960; doi:10.1371/journal.pone.0036992)
Supplement: Table S2 — Summary of mixed effects models for resistance and proportional resistance of aboveground biomass after drought in August 2008 and 2009 to test for effects of diversity (realized numbers of species and functional groups) and management treatments (separated into mowing and fertilizer amounts). (DOC) [file pone.0036992.s006.doc]

Table S1: Summary of mixed effects models for resistance and proportional resistance of aboveground biomass after drought in August 2008 and 2009 to test for effects of diversity (realized numbers of species and functional groups) and management treatments (separated into mowing and fertilizer amounts)

|  |  | Resistance 2008 | | |  | Resistance 2009 | | |  |  | proportional Resistance 2008 | | |  | proportional Resistance 2009 | | |
| --- | --- | --- | --- | --- | --- | --- | --- | --- | --- | --- | --- | --- | --- | --- | --- | --- | --- |
|  | df | AIC | L ratio | *p* |  | AIC | L ratio | *p* |  | df | AIC | L ratio | *p* |  | AIC | L ratio | *p* |
| Nullmodel | 3 | 1026.667 |  |  |  | 1158.463 |  |  |  | 6 | 914.183 |  |  |  | 1025.004 |  |  |
| Block | 6 | 1028.312 | 4.355 | 0.2256 |  | 1157.890 | 6.573 | 0.0868 |  | 9 | 917.457 | 2.726 | 0.4358 |  | 1028.257 | 2.746 | 0.4324 |
| Realized species richness = RSR | 7 | 1027.646 | 2.666 | 0.1025 |  | 1159.533 | 0.358 | 0.5497 |  | 10 | 918.894 | 0.564 | 0.4527 |  | 1030.007 | 0.250 | 0.6170 |
| Realized number of functional groups = RFG | 8 | 1027.727 | 1.919 | 0.1660 |  | 1161.393 | 0.140 | 0.7082 |  | 11 | 918.880 | 2.014 | 0.1559 |  | 1031.973 | 0.034 | 0.8539 |
| Mowing = M | 9 | 1009.877 | 19.850 | <0.0001 | *** | 1147.864 | 15.528 | 0.0001 | *** | 12 | 910.041 | 10.839 | 0.0010 | ** | 1030.268 | 3.705 | 0.0542 |
| M x RSR | 10 | 1007.968 | 3.909 | 0.0480 | * | 1149.831 | 0.033 | 0.8551 |  | 13 | 911.402 | 0.639 | 0.4241 |  | 1032.264 | 0.004 | 0.9492 |
| M x RFG | 11 | 1009.417 | 0.551 | 0.4579 |  | 1150.676 | 1.155 | 0.2825 |  | 14 | 911.165 | 2.237 | 0.1348 |  | 1033.854 | 0.409 | 0.5223 |
| Fertilizer amount = F | 12 | 1010.960 | 0.456 | 0.4994 |  | 1152.372 | 0.304 | 0.5811 |  | 15 | 912.210 | 0.955 | 0.3285 |  | 1035.854 | 0.000 | 0.9860 |
| F x RSR | 13 | 1011.206 | 1.754 | 0.1853 |  | 1153.718 | 0.654 | 0.4187 |  | 16 | 913.842 | 0.369 | 0.5436 |  | 1037.849 | 0.005 | 0.9429 |
| F x RFG | 14 | 1012.844 | 0.362 | 0.5471 |  | 1154.205 | 1.513 | 0.2187 |  | 17 | 915.770 | 0.071 | 0.7892 |  | 1038.507 | 1.342 | 0.2466 |

Models were fitted by stepwise inclusion of variables and likelihood ratio tests (L ratio) were applied to assess statistical significance of variables (p-values). Significance is given with * = *p*<0.05, ** = *p*<0,01, *** = *p*<0.001; df = degrees of freedom.
