# Supplementary material for: Grassland Resistance and Resilience after Drought Depends on Management Intensity and Species Richness
Source: PLoS One. 2012 May 16;7(5):e36992. doi: 10.1371/journal.pone.0036992 (PMC3353960; doi:10.1371/journal.pone.0036992)
Supplement: Table S3 — Summary of mixed effects models for resilience computed as the difference between previously drought and ambient treatment in aboveground biomass as well as for proportional resilience of the first cut in spring 2009 to test for effects of management (separated into mowing and fertilizer amounts) and diversity (realized numbers of species and functional groups) treatments. (DOC) [file pone.0036992.s007.doc]

Table S1: Summary of mixed effects models for resilience computed as the difference between previously drought and ambient treatment in aboveground biomass as well as for proportional resilience of the first cut in spring 2009 to test for effects of management (separated into mowing and fertilizer amounts) and diversity (realized numbers of species and functional groups) treatments.

|  |  | Resilience Spring 2009 | | |  | proportional Resistance 2009 | | |
| --- | --- | --- | --- | --- | --- | --- | --- | --- |
|  | df | AIC | L ratio | *p* |  | AIC | L ratio | *p* |
| Nullmodel | 6 | 3898.242 |  |  |  | 1024.902 |  |  |
| Block | 9 | 3903.177 | 1.065 | 0.7855 |  | 1026.706 | 4.196 | 0.2410 |
| Realized species richness = RSR | 10 | 3904.184 | 0.994 | 0.3188 |  | 1028.635 | 0.070 | 0.7911 |
| Realized number of functional groups = RFG | 11 | 3906.152 | 0.032 | 0.8583 |  | 1030.424 | 0.211 | 0.6456 |
| Mowing = M | 12 | 3905.695 | 2.457 | 0.1170 |  | 1031.792 | 0.632 | 0.4268 |
| M x RSR | 13 | 3907.682 | 0.013 | 0.9078 |  | 1032.456 | 1.336 | 0.2477 |
| M x RFG | 14 | 3909.522 | 0.160 | 0.6894 |  | 1034.034 | 0.422 | 0.5162 |
| Fertilizer amount = F | 15 | 3907.535 | 3.987 | 0.0458 | * | 1034.328 | 1.706 | 0.1914 |
| F x RSR | 16 | 3905.471 | 4.064 | 0.0438 | * | 1035.991 | 0.337 | 0.5615 |
| F x RFG | 17 | 3905.674 | 1.797 | 0.1801 |  | 1037.037 | 0.954 | 0.3288 |

Models were fitted by stepwise inclusion of variables and likelihood ratio tests (L ratio) were applied to assess statistical significance of variables (p-values). Significance is given with * = *p*<0.05, **= *p*<0.01, *** = *p*<0.001; df = degrees of freedom.
